# The role of co-morbidity in the selection of antidiabetic pharmacotherapy in type-2 diabetes

**DOI:** 10.1186/1475-2840-12-62

**Published:** 2013-04-10

**Authors:** Diethelm Tschöpe, Markolf Hanefeld, Juris J Meier, Anselm K Gitt, Martin Halle, Peter Bramlage, Petra-Maria Schumm-Draeger

**Affiliations:** 1 Herz- und Diabeteszentrum Nordrhein-Westfalen in Bad Oeynhausen, Universitätsklinik der Ruhr Universität, Bochum, Germany; 2 Centre for Clinical Studies Professor Hanefeld, Dresden, Germany; 3 Department of Medicine I, St. Josef-Hospital, Ruhr-University Bochum, Bochum, Germany; 4 Institut für Herzinfarktforschung Ludwigshafen, Ludwigshafen, Germany; 5 Zentrum für Prävention und Sportmedizin an der TU München, Munchen, Germany; 6 German Centre for Cardiovascular Research (DZHK), partner site Munich Heart Alliance, Munich, Germany; 7 Institut für Pharmakologie und präventive Medizin, Mahlow, Germany; 8 Med. Klinik 3, Endokrinologie, Diabetologie und Angiologie, Klinikum München-Bogenhausen, München, Germany

## Abstract

Metformin is, if not contraindicated and if tolerated, usually preferred over other antidiabetic drugs for the first line treatment of type-2 diabetes. The particular decision on which antidiabetic agent to use is based on variables such as efficacy, cost, potential side effects, effects on weight, comorbidities, hypoglycemia, risk, and patient preferences. However, there is no guidance how to consider these in the selection of antidiabetic drug treatment. In this work, we aimed to summarize available evidence and tried to give pragmatic treatment recommendations from a clinical practice perspective.

There are clear contraindications for some drugs in those with impaired renal and liver function and precautions in those with heart failure for the use of metformin (NYHA III-IV) and glitazones. On the other hand, GLP-1 analogs, DPP-4 inhibitors and acarbose are generally less critical and can be used in the majority of patients. We identified the following gaps with respect to the selection of antidiabetic drug treatment in patients with co-morbid disease conditions: 1) Guidelines fail to give advice on the use of specific antidiabetic drugs in patients with co-morbidity. 2) The literature is deficient in studies documenting antidiabetic drug use in patients with severely impaired renal function, diabetic retinopathy, cerebrovascular disease and systolic heart failure. 3) Further there are no specific data on patients with multiple of these co-morbid disease conditions. We postulate that differential use of antidiabetic drugs in patients with co-morbid disease constellations will help to reduce treatment related complications and might improve prognosis.

## Introduction

There is appropriate guidance for the pharmacotherapy of patients with type-2 diabetes. In general metformin is, if not contraindicated and if tolerated, considered the first line antidiabetic agent [1]. If non-insulin monotherapy fails in achieving HbA1c targets over 3–6 months, a second oral agent or insulin is to be added. The particular decision on which antidiabetic agent to use is however based on variables such as efficacy, cost, potential side effects, effects on weight, comorbidities, hypoglycemia, risk, and patient preferences.

A recent position statement of the American Diabetes Association (ADA) and the European Association for the Study of Diabetes (EASD) on a patient-centred approach in the management of patients with type-2 diabetes [2] gives an overview on how different glucose-lowering agents may impact treatment choices when co-morbid disease conditions are considered. Even at acceptable levels of glucose control, these comorbidities have a substantial impact on well-being as measured by the SF-36 and EQ-5D [3,4]. Because these considerations are important but difficult to translate into clinical practice, we aimed to summarize available evidence and tried to phrase expert opinion from a clinical practice perspective. We refrained, in general, to comment upon non-antidiabetic drugs as they are beyond the scope of the current review.

**Figure 1:**
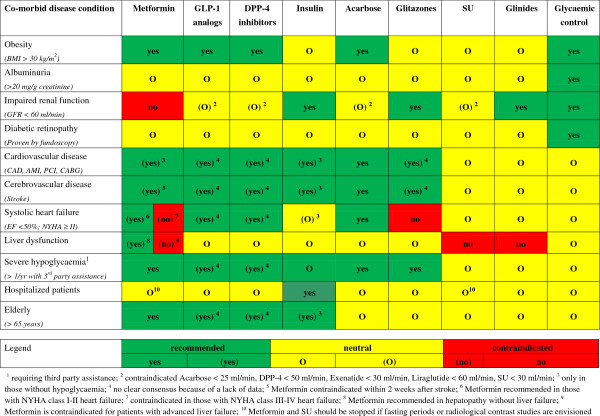
Co-morbidity adjusted selection of antidiabetic drugs based on expert opinion.

This overview was consolidated over the course of a total of two board meetings in which the authors discussed the data on antidiabetic pharmacotherapy in patients with co-morbid disease conditions. On this basis a tabular overview for pragmatic decision making from a clinical practice perspective was developed (Figure 1).

## Antidiabetic therapy in obese patients

In 2010 the prevalence of obesity among adult patients with type 2 diabetes in US was 56.9%, according to the Centres for Disease Control and Prevention. This is not only important on a quantitative basis but also because obesity in patients with diabetes is associated with poor control of blood glucose, making obese patients with diabetes a high risk population for micro- and macrovascular events.

It appears that metformin is the principal choice for obese patients with type-2 diabetes because it is neutral with respect to weight. This has been shown in the *United Kingdom Prospective Diabetes Study* (UKPDS) in which even weight loss was observed for metformin users [5]. On the other hand, the Body Mass Index (BMI) itself appears to have no effect on the reduction of HbA1c or glycaemic control with metformin either alone or in combination with other drugs [5-9], although there are selected data to show that obese patients receiving metformin appear to have a greater chance to achieve target levels of HbA1c < 7% without additional antidiabetic drugs than patients receiving sulfonylurea (SU) or insulin [10]. Dipeptidyl peptidase-4 (DPP-4) inhibitors and bile acid sequestrants are weight-neutral [11,12]. Liraglutide and exenatide have comparable effects on weight, but liraglutide may have a greater effect than exenatide on glycemic control, when used as a second-line therapy [13]. The newer available agents, glucagon-like peptide-1 (GLP-1) agonists promote weight loss. In recent meta-analyses, GLP-1 analogs, were associated with weight loss when added to metformin [11] or to a combination therapy of metformin and SU [14].

Insulin and insulin analogues seem to induce weight gain as a recent meta-analysis showed that insulin and glitazones were associated to weight gain when added to a combination therapy of metformin and SU [14]. A 6-year follow-up of the UKPDS also showed that weight gain was more pronounced in insulin- than in SU- (and metformin-) treated patients [5]. On the other hand, glitazones, SU, and glinides are also associated to weight gain when added to metformin [11]. Despite their effect on weight gain, HbA1c reduction and glycemic control with sitagliptin, nateglinide, glyburide, SU or insulin do not seem to be affected by BMI [5-9].

### Expert opinion

Metformin appears to be the primary choice for obese patients with type-2 diabetes (Figure 1). For metformin-treated patients who fail to achieve targets for fasting plasma glucose of 70 to 130 mg/dl or post-prandial glucose below 180 mg/dl, second-line treatment choices in order of weight benefit would be: GLP-1 agonists, DPP-4 inhibitors, acarbose, bile acid sequestrants, and amylin analogs. If insulin treatment is indicated, a basal insulin supplementation should be considered.

## Antidiabetic therapy in patients with albuminuria

Diabetic nephropathy is characterized by albuminuria and abnormal glomerular function and is a major cause of renal failure. The prevalence of microalbuminuria (MAU) among those with type-2 diabetes was 39% in the *Developing Education on Microalbuminuria for Awareness of Renal and Cardiovascular Risk in Diabetes* (DEMAND) survey [15].

In patients with MAU it has been shown that an intensified glucose lowering treatment is superior to standard of care in reducing levels of MAU [16]. However, data on reduction of albuminuria with particular antidiabetic drugs are scarce: Sitagliptin was shown to reduce MAU in a small pilot study in 36 patients with type-2 diabetes [17]. This was most likely depending on risk factor control such as, among other undetermined factors, blood glucose, blood pressure and inflammation reduction. For glitazones (rosiglitazone), there is a subanalysis of the *A Diabetes Outcomes Prevention Trial* (ADOPT) trial, in which 4,351 recently diagnosed, drug-naïve patients with type-2 diabetes were treated and followed for up to 5 years with rosiglitazone, metformin, or glyburide [18]. While the albumin/creatinine ratio (ACR) rose slowly with metformin it fell with rosiglitazone (and less so with glyburide) within the first 2 years, but slowly increased during the following years. Blood pressure with rosiglitazone was reduced compared to the other treatment options. The data contradict a previous study, however, which compared rosiglitazone versus glyburide for the reduction of urinary albumin excretion, where no significant difference in the reduction of baseline albuminuria between the two drugs was observed [19].

The most relevant study related to MAU is probably the Steno-2 study, which showed that intensive therapy -consisting on tight glucose regulation, lifestyle modification, and the use of the renin-angiotensin system blockers, aspirin, and lipid-lowering agents- had sustained beneficial effects with respect to vascular complications, renal disease, and on total and cardiovascular death in type 2 diabetes patients with persistent MAU [20].

### Expert opinion

No firm recommendations can be derived based on the available literature (Figure 1). The only justified recommendation seems to be a multifactorial intensive intervention to control glucose more tight than in those without albuminuria.

## Antidiabetic therapy in patients with impaired renal function

Diabetes is the leading cause of kidney failure, accounting for 44% of all new cases of kidney failure in 2008 in the United States [21]. The clearance of antidiabetic drugs is decreased and results in prolonged exposure to higher levels of the drug or its metabolites that may trigger adverse events. Observational data suggest that mortality risk in patients with type 2 diabetes and renal dysfunction increases with HbA1c levels < 6.5% and > 8.0%. Accordingly, HbA1c levels between 6.5% and 7.5% may be a reasonable target for these patients [22].

A number of antidiabetic drugs are contraindicated in patients when glomerular filtration rate (GFR) falls below variable thresholds, such as 25 ml/min for acarbose, 30 ml/min for exenatide and SU, 50 ml/min for DPP-4 inhibitors and 60 ml/min for metformin and liraglutide. There are scant data on the actual efficacy and safety of diabetes medications depending on patients’ renal function. Furthermore, contraindications do not necessarily match with available evidence. This is exemplified with metformin [23]: The label of metformin in the U.S. allows the prescription of metformin at or above 1.4 mg/dl serum creatinine in women and 1.5 mg/dl in men. In Germany, metformin is contraindicated in patients with a GFR < 60 ml/min. In the U.K. prescribing guidelines consider both creatinine and GFR for assessing treatment eligibility. The National Institute for Health and Clinical Excellence (NICE) recommends reviewing prescriptions when serum creatinine exceeds 1.5 mg/dl or GFR falls below 45 ml/min per 1.73 m^2^ and to stop treatment at a serum creatinine of 1.7 mg/dl or a GFR of below 30 ml/min per 1.73 m^2^. The uncertainty is mirrored in clinical practice when metformin is prescribed despite full knowledge of the relevant cut-offs. Nevertheless, there is no evidence from prospective comparative or observational studies that metformin confers an increased risk of lactic acidosis. This was shown in a meta-analysis from 347 comparative trials and cohort studies [24].

Insulin is generally considered to be safe in patients with a reduced kidney function. When there is a reduction of renal function, its half-life is prolonged because of lower levels of degradation [25]. This has been shown to increase the frequency of hypoglycaemic events which may be five times as frequent as in patients without kidney disease [26].

Available pharmacokinetic and clinical studies suggest that DPP-4 inhibitors may be safe in patients with renal impairment. Because of their variable degree of renal clearance (except for linagliptin), they may however need dose adjustment. Additionally, DPP-4 inhibitors have a decreased risk of hypoglycemia [27].

As outlined above, glitazones may have a protective effect to either prevent or slow the progression of diabetic kidney disease. This drug class may therefore be preferred over other drugs for the treatment of patients with diabetes. It further undergoes hepatic metabolism and has been demonstrated to be effective without an increased risk of hypoglycemia in patients with chronic kidney disease [28]. However, fluid retention may be accentuated and appropriate measures should be taken.

### Expert opinion

Impairment of renal function is an important co-morbidity to be considered when selecting antidiabetic drugs (Figure 1). When taking a GFR < 60 ml/min metformin is contraindicated as is liraglutide. Other thresholds apply to DPP-4 inhibitors (<50), exenatide and SU (<30) and acarbose (<25 ml/min). Generally recommended are glitazones, glinides, insulin, and more recently DPP-4 inhibitors, although the dose may have to be adjusted.

## Antidiabetic therapy in patients with diabetic retinopathy

Diabetic retinopathy is the most frequent vascular complication and the most feared by patients. The prevalence ranges in newly diagnosed patients ranges from 0 to 30% [29]. Diabetic macular edema (DME) was present in 4.1% of type-2 diabetic patients not requiring insulin and 9.1% in those with insulin in the longitudinal Exeter Diabetic Retinopathy Screening Program in the UK [30,31].

Optimal glycaemic control is crucial for preventing the progression of retinopathy. Furthermore, an early achievement of good glycaemic control in the course of diabetes has been shown to delay progression of diabetic retinopathy independent of glucose control [32]. The use of retinal laser photocoagulation together with intravitreal therapy with steroids, and more recently, with biologicals has enabled a second line therapy for DME [33].

Among the antidiabetic drugs insulin has been shown to directly influence retinal blood flow, vascular tone and angiogenesis [34]. For glitazones, the UKPDS demonstrated for each 1% reduction in HbA1c level a corresponding 31% reduced risk of onset or progression of diabetic retinopathy over 9 years of follow-up [32]. Nevertheless, TZDs have been reported to increase the risk of DME in a prospective cohort study [35], but data from a subgroup of the *Action to Control Cardiovascular Risk in Diabetes* (ACCORD) eye substudy did not demonstrate a conclusive link between TZD use and DME [36].

### Expert opinion

No conclusive recommendations can be derived based on the available literature (Figure 1). All agents are about as good for patients with diabetic retinopathy. Since the Steno-2 study showed that intensive therapy significantly reduced the risk of retinopathy by about 60% in type-2 diabetes patients with MAU [20], it seems justified an intensive therapy to control glucose more tight than in those without retinopathy.

## Antidiabetic therapy in patients with cardiovascular disease

Coronary artery disease (CAD) is the most common cause of morbidity and mortality in patients with type-2 diabetes. In 2004, heart disease was noted on 68% of diabetes-related death certificates among people aged 65 years or older in the United States [21].

It has been postulated that metformin might promote CAD [37] and is known to bear the additional risk of lactic acidosis, especially in patients with recent myocardial infarction [38]. Fisman et al. even found an increase of mortality in CAD patients receiving metformin after a 5-year follow-up [39]. These findings should, however, be considered with caution, since the study was non-randomized. A direct comparison of metformin with other antidiabetic drugs is only available from two retrospective cohort studies. The first included 8,494 patients after myocardial infarction in Denmark and found lower mortality rates for metformin than for SU users and risk appeared to be increased in men [40]. The second included patients with a prior diagnosis of ischemic heart disease treated with metformin and documented a lower all-cause mortality for metformin than for either SU or repaglinide [41]. Nevertheless, a recently published meta-analysis could not demonstrate the benefit/risk ratio of metformin [42]. Therefore, further studies are warranted to clarify the effects of metformin on cardiovascular mortality and morbidity among patients with type 2 diabetes.

A meta-analysis of 35 trials including 8,478 patients examined the effect of insulin on mortality in the hyperglycemic critically ill patient, mostly after myocardial infarction [43]. It demonstrated that insulin decreased short-term mortality by 15%. A considerable benefit was noted in patients with type-2 diabetes mellitus (RR 0.73; 95%CI 0.58-0.90) [44]. In the Diabetes Mellitus Insulin Glucose Infusion in Acute Myocardial Infarction 2 (DIGAMI 2) trial glucose-insulin infusion failed to result in a survival benefit however [45].

Acarbose is the most extensively studied alpha-glucosidase inhibitor. In STOP-NIDDM subjects with prediabetes and early diabetes were included and suggested that acarbose was associated with a 49% risk reduction in cardiovascular events [46].

SU, on the other hand, have been reported to reduce myocardial blood flow, increase infarct size, and to increase early mortality after direct angioplasty for acute myocardial infarction. These untoward effects have been reported more so for the first-generation SU such as tolbutamide [47] than for second-generation compounds. However, the aforementioned Danish registry found no difference with respect to the type of SU as to 30-day and 1-year mortality [48]. Importantly, the most recent data on the Danish registry on SU suggests that there is not a class effect on mortality and cardiovascular risk [49]. The study showed that monotherapy with the most used SU, including glimepiride, glibenclamide, glipizide, and tolbutamide, seemed to be associated with increased mortality and cardiovascular risk compared with metformin. Similar to SU, glinides modify ATP-dependent potassium channels and therefore should be used with caution.

### Expert opinion

In patients with cardiovascular disease, SU or glinides should rather be avoided and other treatment options preferred instead (Figure 1). Because of the risk of hypoglycemia blood glucose targets to be met are suggested to be less tight compared to patients without cardiovascular disease.

## Antidiabetic therapy in patients with cerebrovascular disease

In 2004, stroke was noted on 16% of diabetes-related death certificates among people aged 65 years or older in the United States and the risk for stroke is 2 to 4 times higher among people with diabetes [21]. When considering antidiabetic treatment in stroke patients three different situations have to be considered. 1) Preadmission use of antidiabetic drugs; 2) antidiabetic drug use after acute stroke and 3) antidiabetic drug use in patients with anamnestic stroke.

1) In a Danish follow-up study of 4,817 patients hospitalized with ischemic stroke between 2003 and 2006 [50] the preadmission use of metformin, insulin, and patients without antidiabetic pharmacotherapy had a lower 30-day mortality compared with users of SU. However, no significant differences were observed in 1-year mortality rates. TZDs were shown to reduce infarct volume and improve neurologic function following transient middle cerebral artery occlusion in rats [51].

2) Antidiabetic drugs during acute stroke are based on the association of hyperglycemia with poor outcome after acute ischemic stroke. The 2007 American Heart / American Stroke Association guidelines suggest treating at lower glucose levels based on expert opinion [52]. One of the few studies investigating pharmacotherapy in this situation was the Glycemia in Acute Stroke study (GLIAS), a multicenter, prospective, and observational cohort study of 476 acute ischemic stroke patients [53]. Capillary blood glucose ≥155 mg/dl was associated with a 4-fold increase in the odds of poor outcome at 3 months after adjustment for age, gender, hypertension, diabetes, stroke severity, admission glycemia, and infarct volume. The UK Glucose Insulin Stroke Trial (GIST-UK) suggests that most acute stroke patients will only have mild to moderate increases in plasma glucose at presentation (median 7.6 mmol/l) with minimal insulin requirement as a consequence [54]. Metformin is contraindicated in patients within 2 weeks after stroke.

3) We found no data to prefer any antidiabetic drug over another in patients with anamnestic stroke.

### Expert opinion

Recommendations for those with cerebrovascular disease in general match those with cardiovascular disease (Figure 1). While SU and glinides should be avoided, all other treatment options can be used to control blood glucose as appropriate.

## Antidiabetic therapy in patients with (systolic) heart failure

Patients with type-2 diabetes are at high risk for developing systolic heart failure. In a population based cohort (Saskatchewan Health database, Canada) of patients with recent-onset type-2 diabetes the incidence was 6% over 5.2 years [55]. Pharmacotherapy is particularly difficult in this patient group as anti-diabetic medication may impair myocardial energy metabolism, thus influencing symptoms and clinical outcome. Although metformin is contraindicated in these patients in some countries, a recent systematic review by Eurich revealed that treatment with metformin may be associated with lower mortality rates, lower rates of all cause hospital admission, and less adverse events [56]. Data from the UKPDS showed that the risk for heart failure was not significantly different between the groups treated with metformin and its controls [57]. Furthermore, a study conducted in Denmark prospectively followed 10,920 patients hospitalized for the first time for heart failure in 1997–2006 and who were receiving metformin, SU and/or insulin. The study showed that treatment with metformin was associated with a low risk of mortality in diabetic patients with heart failure compared with treatment with SU or insulin [58].

Data available for acarbose in patients with heart failure are limited. In a meta-analysis of seven randomized, controlled studies with a minimum treatment duration of 52 weeks in type-2 diabetic patients and no heart failure at baseline incident heart failure was rare (about1%) and there was a trend towards a reduced incidence of heart failure with acarbose (HR 0.55 (95%CI 0.21-1.45) [59]. Insulin has been found to have positive inotropic effects on myocardial tissue and improved hemodynamics in patients with systolic heart failure. A retrospective cohort study in 16,417 patients with diabetes and a primary diagnosis of heart failure found no association between the use of insulin and mortality in comparison to other antidiabetic drugs [60].

### Expert opinion

It appears that glitazones and, in NYHA III-IV heart failure, metformin should not be used in patients with heart failure (Figure 1). Other drugs such as insulin, SU and glinides are less likely to produce untoward effects. Generally preferred are drugs such as metformin (NYHA I-II), acarbose and GLP-1 analogs / DPP-4 inhibitors although the evidence base for the recommendation of the latter is weak.

## Antidiabetic therapy in patients with liver dysfunction

Liver dysfunction, particularly non-alcoholic fatty liver disease (NAFLD), and type 2 diabetes frequently coexist. Indeed, 49–62% of type 2 diabetes patients have NAFLD [61]. NAFLD may be a marker of cardiovascular risk and mortality in type 2 diabetes patients.

Treatment of type 2 diabetes in patients with liver dysfunction is complex as many OADs may be contraindicated because the risk of hypoglycemia may be magnified, and therefore doses may also need to be adjusted. There are only a few reported cases of hepatotoxic side effects for metformin [62], but there may be an increased risk of developing lactic acidosis in the setting of impaired liver function. Therefore, metformin is contraindicated for patients with advanced liver disease. Nevertheless, the risk of lactic acidosis associated with metformin seems to accentuate in patients with multiple comorbidities, particularly when there is an acute deterioration [63]. If a patient has stable liver dysfunction and few comorbidities, metformin is likely to be reasonably safe, but the dose should be reduced to 1500 mg daily maximum and it should be discontinued if renal or liver function worsens [64-66].

Insulin therapy is probably the safest and most effective therapy in patients with liver dysfunction, with the limitation that increased risk of hypoglycemia in such patients requires extra care with insulin dosage. Regarding SU and glinides, the existence of increased risk of hypoclycemia due to reduced hepatic gluconeogenesis should also be considered. Indeed, glinides are contraindicated in the setting of liver dysfunction because they are mostly cleared through the liver, and SU are contraindicated in advanced liver failure.

Data regarding the use of DPP-4 inhibitors in patients with liver disease are currently lacking.

Patients with moderate liver dysfunction can take sitagliptin or exenatide [67,68]. Vildagliptin is contraindicated in patients with liver dysfunction as clinical studies showed that high doses of this drug increased hepatic transaminases in some patients [69].

### Expert opinion

Type 2 diabetes patients with liver dysfunction are at increased risk of cirrhosis, liver failure and hepatocellular carcinoma. Identification and tight control of metabolic risk factors remains the mainstay of treatment. Although based on the available literature no conclusive recommendations can be derived, given that OADs are contraindicated in patients with advanced liver disease, insulin treatment remains the available option for glycemic control.

## Antidiabetic therapy in patients with episodes of severe hypoglycaemia

Hypoglycemia is the principal reason that blood glucose targets are not achieved in many patients and has been reported to occur at 10 to 70 per 100 patient years [70]. This is associated with an increased rate of cardiovascular and total mortality [71]. While acarbose, metformin, pioglitazone, and incretine-based therapies are not associated with hypoglycemia, a mono- or combination therapy with insulinotropic OADs such as SU or glinides and insulin have been associated with an increased risk. In the UKPDS 16% of SU and 36% of insulin treated patients experienced hypoglycemia [57]. A later study reported substantially higher rates for glibenclamide (38%) [72] which would alleviate the difference between SU and insulin [70]. Furthermore, the risk of hypoglycemia is strongly dependent on the choice of insulin. Long-acting (analog) insulins have a lower propensity to induce severe hypoglycemia compared to neutral protamine Hagedorn (NPH) insulin. Regarding short acting insulins, a meta-analysis was conducted to assess its effects compared to regular human insulin [73]. Their results suggested only a minor benefit of short acting insulin analogues in terms of incidence of hypoglycemic events.

### Expert opinion

In patients with a history of (severe) hypoglycemia drugs such as SU, glinides and insulin should be avoided and less stringent blood glucose targets pursued (Figure 1). On the other hand, metformin, GLP-1 analogs / DPP-4 inhibitors, acarbose and glitazones appear to confer a low risk and should be preferred.

## Antidiabetic therapy in hospitalized patients

Diabetes is associated with increased hospital admission and length of hospital stay [74] and there is much evidence that type 2 diabetes increases inpatient morbidity and mortality [75,76]. Given the particular conditions of inhospital patients, their treatment requires more flexible strategies to treat hyperglycemia [69].

Current guidelines for inhospital patients with diabetes recommend that the glucose concentration target value should be maintained in the 140 to 180 mg/dL range as long as these values can be achieved safely [77]. Lower targets can also be considered but it is of utmost importance to avoid hypoglycemic events. However, one should bear in mind that the effect of hyperglycemia and mortality rate among patients with diabetes in surgical, medical and cardiac intensive care units depends on the admission diagnosis, as shown in a study by Falciglia et al. [78].

Insulin is considered the standard of inpatient glucose management [77]. Nevertheless, if the hospital stay is expected to be short and metabolic derangements are unlikely to occur, patients may continue their home regimen. After fasting periods, OADs should be only continued after the first normal meal. Indeed, there are special recommendations for SU and metformin. Temporarily discontinuing treatment with SU is recommended if fasting periods are expected due to increased risk of hypoglycemia [79]. Metformin should be discontinued 48 hours before the intended procedure and not resumed until the patient tolerates oral food intake and there are no other contraindications. Insulin can be administered when OADs are temporarily discontinued. Metformin is also contraindicated in contrast studies as iodinated radiological contrast media may lower the glomerular filtration rate with the risk of causing renal failure. Therefore, metformin should be discontinued at least 24 hours before the contrast study and restarted 48 hours afterward if normal renal function is confirmed. Similarly, SU should be also temporarily stopped before radiological tests, as they are mainly cleared through the kidneys. On the other hand, it should be also considered that high-dose glucocorticoid treatment increments the insulin requirement. Specifically, insulin dose should be adjusted postprandially and 8 to 12 hours after glucocorticoid administration [79].

### Expert opinion

In view of the many restrictions on the use of oral antidiabetic drugs, temporary insulin treatment remains the most practical means of glycemic control for many hospitalized patients with type 2 diabetes. Glucose-lowering strategies must be chosen individually for each patient, with consideration of the relevant comorbidities and special conditions.

## Antidiabetic therapy in elderly

The majority of patients have to be considered “elderly” when an age above 65 years is used as a threshold. “The elderly” however is a heterogeneous group with varying physiologic profiles, functional capabilities, and life expectancy. While some are actually “young”, many suffer from multiple co-morbidities for which specific considerations have been summarized above [80].

The risk of hypoglycemia is increased in elderly patients due to impaired renal and hepatic function, drug interaction and malnutrition [81] and a loss of sensitivity towards hypoglycemia has been reported [82]. Thus, HbA1c targets can, correspondingly, be less stringent [83]. Furthermore, a proper balance of lowering blood glucose and not getting too low is difficult to accomplish in the elderly. This is particularly true for SU in which the risk of hypoglycemia is increased by 36% in the elderly compared with younger adults.

Substantial gastrointestinal side effects may limit the use of alpha-glucosidase inhibitors particularly in older patients. There is limited information on the use of DPP-4 inhibitors in the elderly. DPP-4 inhibitors may, however need their dose adjusted (see section on nephropathy). There are not sufficient data on exenatide use in the elderly. It may be beneficial in elderly patients with reduced mobility who could benefit from weight loss, whereas it is less optimal for frail, normal weight adults. However, trials in the elderly have not been performed in larger cohorts.

A recent study investigated the efficacy and safety of adding once-daily insulin glargine to patients’ current OAD treatment and compared this to a strategy of increasing OAD doses in the elderly with poor glycemic control [84]. During the observation, HbA1c was reduced by 1.5% in insulin treated vs. 0.6% in OAD treated patients. Mean fasting blood glucose decreased by 29 and 15% and insulin treated patients had fewer hypoglycemic events. The results suggest that the addition of insulin glargine to oral antidiabetic drugs is safer with respect to hypoglycemia but as effective in elderly patients.

### Expert opinion

Age is not a co-morbid disease condition per se but usually a constellation that increases the likelihood of co-morbid diseases such as those outlined above. Therefore most of the treatment recommendations for the elderly have to rely on other co-morbidities (Figure 1). Preferred drugs in the older patients should, ideally, not be cleared by the kidney and have a low risk of adverse cardiovascular effects or hypoglycaemia. Generally metformin, GLP-1 analogs / DPP-4 inhibitors and long-acting insulin should be preferred over other treatment options (SU, glinides and glitazones).

## Perspectives

Randomized, controlled trials often employ narrow inclusion criteria, enrolling patients at a low risk for complications, a low degree of co-morbidity and to avoid the enrollment of patients with adverse effects or poor adherence. We identified the following gaps with respect to the selection of antidiabetic drug treatment in patients with co-morbid disease conditions: 1) Guidelines fail to advice on the use of specific antidiabetic drugs in patients with co-morbidity. 2) The literature is deficient in studies documenting antidiabetic drug use in patients with severely impaired renal function, diabetic retinopathy, cerebrovascular disease and systolic heart failure. 3) Further there are no specific data on patients with multiple of these co-morbid disease conditions.

## Competing interests

Basis for the preparation of this article were two Advisory Board Meetings. For the participation at these board meetings members received financial compensation by Sanofi Aventis Deutschland GmbH. The authors were free in the selection of content and the decision to publish the results. They take full responsibility for the content of this article. Diethelm Tschöpe and Peter Bramlage serve as guarantors. DT, MH, JJM, AKG, MH, PB and PMSD received consultancy fees, attended advisory boards and have held lectures for a number of pharmaceutical companies producing antidiabetic drugs including sanofi-aventis.

## Authors’ contributions

The present manuscript has been developed over the course of a total of two board meetings in which all authors gathered and discussed the data on antidiabetic pharmacotherapy in patients with co-morbid disease conditions. PB assembled the manuscript and screened the literature for further supporting evidence. All authors revised the article for important intellectual content. All authors read and approved the final manuscript.
